# Evaluation of training models for intraventricular neuroendoscopy

**DOI:** 10.1007/s10143-024-03082-9

**Published:** 2024-11-11

**Authors:** Sebastian Senger, Magomed Lepshokov, Thomas Tschernig, Guiseppe Cinalli, Joachim Oertel

**Affiliations:** 1 Department of Neurosurgery, Medical School of Saarland University, Homburg/Saar, Germany; 2 Institute of Anatomy and Cell Biology, Medical School of Saarland University, Homburg/Saar, Germany; 3 Department of Pediatric Neurosurgery, Santobono-Pausilipon Children’s Hospital, AORN, Naples, Italy; 4 Universität des Saarlandes, Kirrberger Straße 1; Gebäude 90.5, 66424 Homburg, Germany

**Keywords:** Neuroendoscopy, Surgical education, Murine model, Cadaver model, Endoscopy

## Abstract

Structured surgical education has become increasingly important in recent years. Intraventricular neuroendoscopic procedures have been widely established. However, training surgical skills with these techniques is crucial for young residents due to the potential harm to adjacent tissue. Therefore, we evaluated two different training models. Participants in two different international workshops were trained on a prefixed cadaver model and on a living murine intraabdominal model. Crucial neuroendoscopic techniques such as membrane perforation and tissue biopsy were performed. A blinded questionnaire evaluated both models. Sixty-three participants were trained on the animal model. Forty of these were trained on the cadaver model. The training effect was evaluated almost equally, with 8.5/10 for the animal model and 8.9/10 for the cadaver model. The tissue properties were rated higher regarding realism in the animal model, whereas the anatomic realism was rated higher in the cadaver model. The animal model is a valid alternative to cadaver models for teaching endoscopic neurosurgical skills. This model benefits from the simulation of real surgical tissue properties, including bleeding. The low costs and availability of this technique make it more ubiquitous and can help train further generations of neurosurgeons.

## Introduction

The first neuroendoscopic procedures were performed more than 100 years ago but soon stopped due to technical and medical limitations [1]. Neuroendoscopy was revolutionized after the collaboration of Harold Hopkins and Karl Storz in the 1970s [2]. Endoscopic techniques were restricted to a few specialized centers until the end of the 1980s when well-known neurosurgeons began to take this technique seriously into account [3].

Endoscopic treatment options include the restoration of the physiological pathway, e.g., with foraminoplasty, or the opening of alternative pathways through fenestrations, e.g., ETV, fenestration of the lamina terminalis or septostomy. In the case of mass lesions, the removal of the lesion must be considered [4–7].

**Fig. 1:**
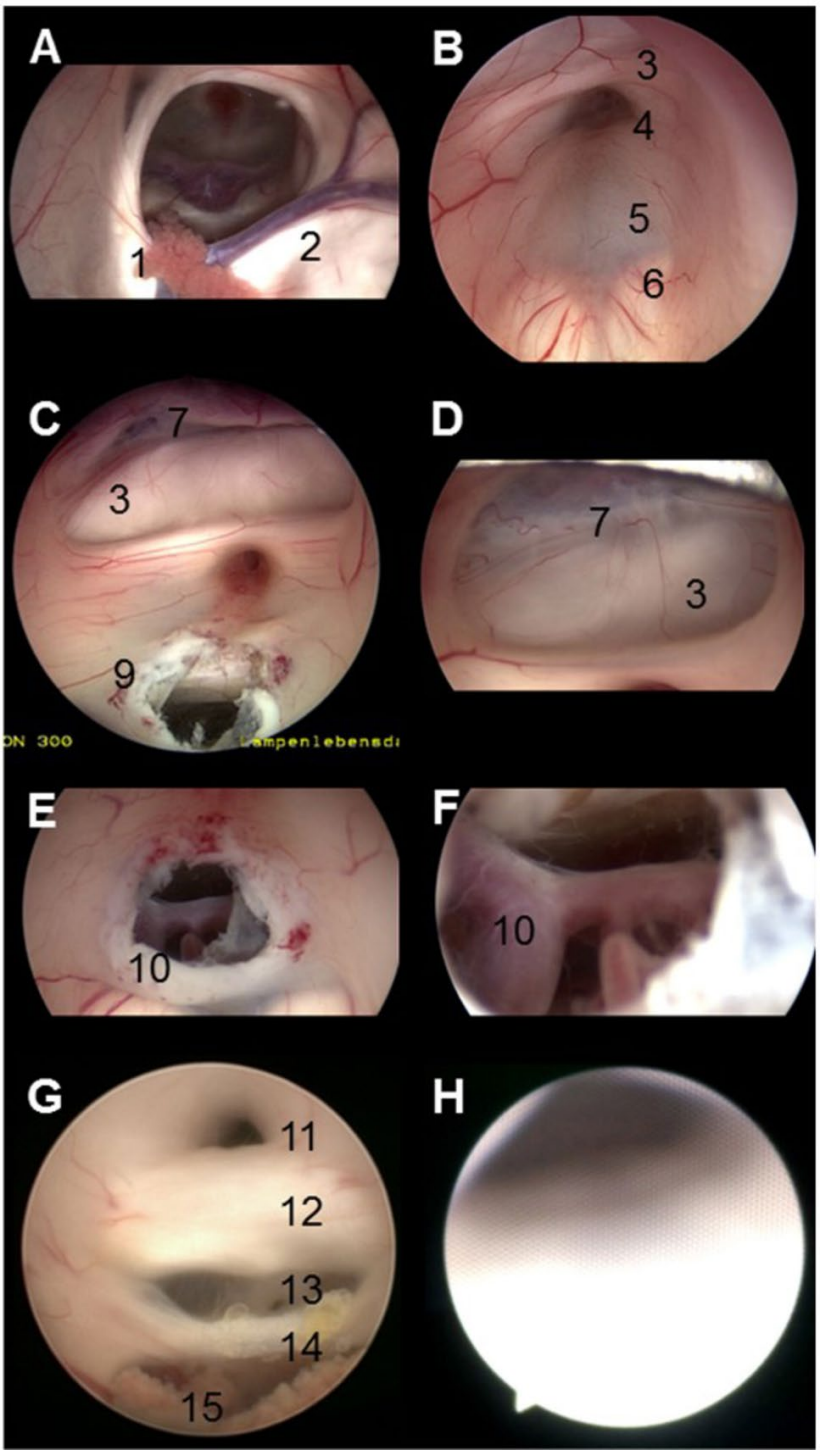
Intraoperative screenshots of the ventricular system. Endoscopic view with the 0-degree optic inside the right lateral ventricle. The right foramen of Monro is in the center. The choroid plexus (1) runs through it to the roof of the 3rd ventricle. The thalamostriate vein (2) runs on the right. The fornix forms the medial and upper parts of the foramen. The thalamus forms the lateral part. The 3rd floor can be identified through the foramen of Monro (**A**). Viewing through the 3rd ventricle, the endoscope was carefully moved through the foramen. The optic chiasm can be identified at the top (3), followed by the infundibular recess (4), the clivus at the floor (5) and the mammillary bodies (6) (**B**). The 3rd ventricle was viewed after ETV. The lamina terminalis (7) and the optic chiasm (3) can be identified. The bony clivus can be seen through the newly formed stoma (9) (**C**). A 30-degree optic view was used for comparison. The lamina terminalis, optic chiasm and anterior commissure can be identified more precisely (**D**). Screenshot of the stoma after ETV with identification of the basilar artery (10) (**E**). Inspection through the stoma to ensure communication of the CSF and exclude Lillequist’s membrane (**F**). The posterior part of the third ventricle was viewed using angled optics. The triangularly shaped sylvian aqueduct (11), posterior commissure (12), pineal body (13), habenular commissure (14) and choroid plexus (15) can be identified (**G**). A flexible endoscope was used to view the entrance of the aqueduct (**H**)

 The goal of international societies is to promote these minimally invasive techniques and teach their application in low-income countries, for instance. However, neuroendoscopy differs slightly from other endoscopic procedures in other surgical fields. General surgeons, orthopedics or urologists, for example, have implemented endoscopic procedures in their daily practice, including different levels of difficulty. Arthroscopic or laparoscopic procedures are well established, and physicians start practicing these procedures early during residency. Courses and training models are available in large numbers. Moreover, the indications for an intraventricular neuroendoscopic procedure are rather rare or are only frequently performed in specialized centers, for instance, for pediatric neurosurgery, or in larger departments with a high number of cases per year. Therefore, compared with other young surgeons, residents might not have the chance to see and practice endoscopic procedures. Although intraventricular procedures are mostly straightforward short procedures and can be performed by inexperienced surgeons under the instruction of an experienced neuroendoscopist, some limitations must be mentioned. First, the knowledge and handling of the endoscope and the instruments must be understood. As mentioned, it might be unfamiliar to young neurosurgeons to look at the screen and not through the microscope. Second, the space in the ventricle system might be limited, and maneuvers can be challenging. Third, the structures adjacent to the ventricle system are very eloquent. Accidental injuries to these structures might cause permanent deficits such as short-term memory deficits (due to Fornix lesions), eye muscle palsy (due to oculomotor nerve lesions) or even hemiparesis (due to thalamic lesions). Injuries to vessels such as the thalamostriate vein or even the basilar artery can result in severe bleeding and even death, as previously reported (Fig. 1) [8–11]. These injuries can occur not only by improper instrument handling but also, for example, by incorrect coagulation and thermal damage. This emphasizes how important the teaching of these techniques is to avoid complications for the patient. The logical alternatives to train neuroendoscopic procedures on patients are models and simulations. Concerning the current status of neurosurgical models with other surgical fields, we performed a PubMed search for the “Laparoscopic training model”, “Arthroscopic training model” and “neuroendoscopic training model”. This simple keyword search revealed 21,537 papers concerning laparoscopic training models, 5656 papers concerning arthroscopic training models and only 194 papers concerning neuroendoscopic training models. These results emphasize how underrepresented this topic is in the neurosurgical community.

The authors have established annual workshops for neuroendoscopy training over the last 15 and 12 years in two neurosurgical departments. These workshops include lectures, live surgeries and practical hands-on sessions. One model is based on a murine model for the simulation of intraventricular procedures. It was introduced by the authors from the very first edition of their workshop more than 15 years ago, who recalled the experience of Professor Jacques Camaert, who first introduced this model to train in neuroendoscopic procedures in his workshops held for almost 15 years in Ghent, Belgium, until the last edition in 2012. To the best of our knowledge, he has never described or reported his model in the literature. Since then, it has been adopted and described in the literature [12, 13].

Given the potential risk of an intraventricular procedure, models simulating this environment might not cover all aspects, such as bleeding or tissue properties. With a live animal model, these aspects should be simulated as intended.

The use of animal models is under constant debate, and animal protection laws in many countries encourage scientists and physicians to refine, reduce and/or replace animal experiments. Therefore, the aim of the present study was to evaluate the murine model in comparison to other models on the learning effect of trainees.

## Materials and methods

### Animal model

The procedures were performed under approval by the local governmental animal care committee (registration number TVA 11-2023) and were in accordance with the UKCCCR Guidelines for the Welfare of Animals in Experimental Neoplasia (Br J Cancer 1998; 77:1–10) and the Interdisciplinary Principles and Guidelines for the Use of Animals in Research (New York Academy of Sciences Ad Hoc Committee on Animal Research, NY). Sprague–Dawley rats with a minimum weight of 250 g were obtained from Charles Rivers Laboratories, Sulzfeld, Germany. The animals were housed in cages at a room temperature of 22–24 °C and a relative humidity of 60–65% with a 12-h light/dark cycle. The rats were allowed free access to drinking water and standard laboratory chow (Altromin^®^, Lage, Germany). The animals were anesthetized by initial inhalation of isoflurane. Anesthesia was induced by intraperitoneal injection of 90 mg/kg bodyweight ketamine (Ketavet^®^, Parke Davis; Freiburg, Germany) and 8 mg/kg bodyweight xylazine (Rompun^®^, Bayer; Leverkusen, Germany). All animals received 5 mg/kg body weight carprofen (Vetranal™, Sigma‒Aldrich; St. Louis, USA) subcutaneously in addition to pain relief.

**Fig. 2:**
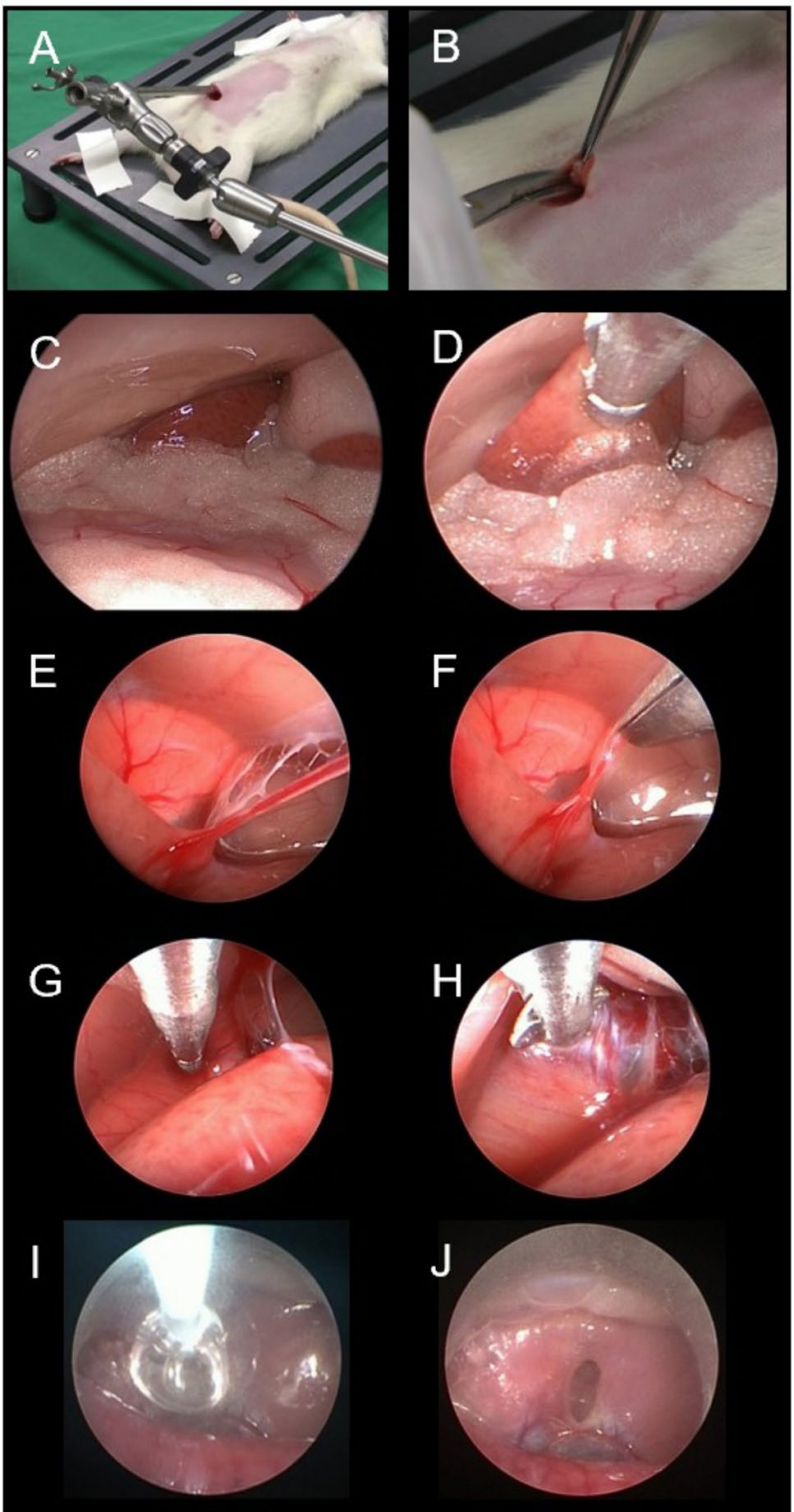
Overview of the murine model. The rat was placed in the supine position (**A**), and the abdominal wall was opened (**B**). The liver was identified (**C**). The biopsy was performed with biopsy forceps (**D**). The falciform ligament is identified (**E**) and cut with scissors (**F**). The diaphragm was identified and perforated with perforating forceps (**G**, **H**). The balloon catheter was inflated, and the stoma was enlarged (**I**). The stoma can be inspected, and the procedure ends with a lethal dose of pentobarbital (**J**)

 The animals were fixed in the supine position, and the limbs were fixed with tape. The anesthesia was constantly evaluated, and additional ketamine was applied if necessary. A median skin incision was made to access the peritoneal space (Fig. 2). An endoscope was inserted, and the skin was closed with a circular suture. There are two options: the model can be applied under water and constant irrigation with Ringer’s solution or under air conditions. The authors suggest the use of antifogging fluids to avoid forging of the lenses if the second method is used. The surgical steps were demonstrated to the trainees by the tutor on videos or pictures before the surgery. The following surgical steps were performed by the trainee.

First, the endoscope is introduced, and an inspection of the abdominal space is performed. Second, the liver lobes are identified, and coagulation at the rim of the liver lobe is performed. Then, the coagulated tissue was removed with biopsy forceps. Possible bleeding from the parenchyma can be stopped with a bipolar probe.

After this step, the endoscope can be rotated 180 degrees to expose the bladder. This procedure simulates tumor removal. The attaching ligaments can be cut with scissors, and the bladder can be removed with grasping forceps.

Then, the endoscope is turned again and moves up to the upper part of the liver and the diaphragm. As a next step, the ligamentum falciforme is exposed and can be cut.

The last step is fenestration of the diaphragm. Here, coagulation is performed, the diaphragm is perforated with blunt perforating forceps, and the stoma is enlarged with balloon catheters. This simulates an ETV. The animal was then euthanized with a lethal dose of pentobarbital as it otherwise died due to collapse of the lung.

### Cadaver model

**Fig. 3:**
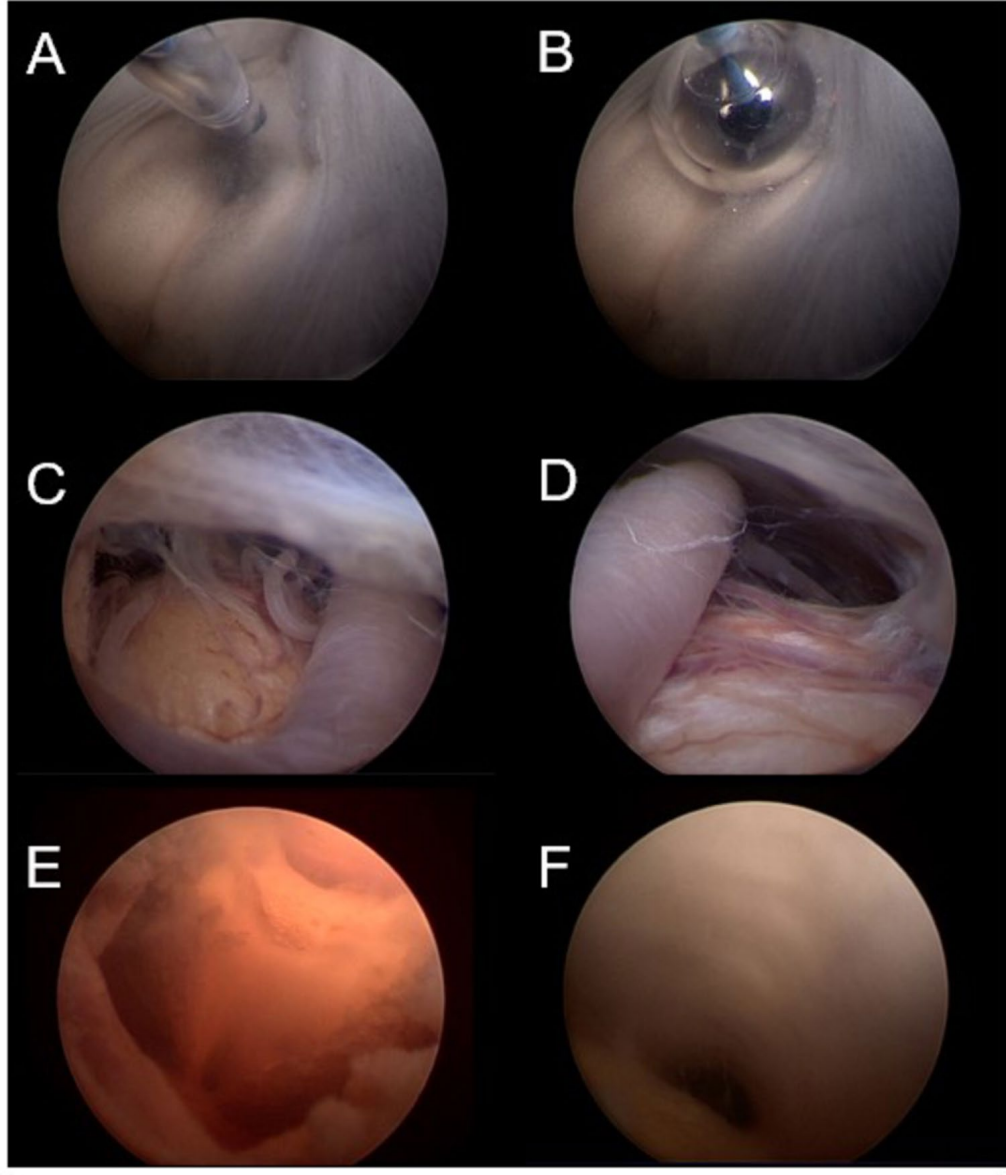
Overview of the cadaver model. After the trocar is introduced, the third ventricle is identified, and perforation is performed between the mammillary bodies and the infundibular recess (**A**). Enlargement of the stoma with the balloon catheter (**B**). The stoma was viewed to identify the basilar artery and perforating vessels on both sides (**C**, **D**). Inspection of the dorsal part of the third ventricle with the entrance of the aqueduct (**E**). Visualization of the triangular-shaped aqueduct (**F**)

 The endoscopic procedures can also be trained on cadaver models. The Institute of Anatomy and Cell Biology of the Saarland University provides the human cadavers. The education of physicians was performed under approval by the local governmental ethic committee (registration number 245/22). In general, these fresh frozen or prefixed cadavers allow surgical procedures, including borehole trepanation, endoscopic puncture of the ventricle and inspection and perforating steps (Fig. 3). The trainees were instructed to perform a ventricle puncture 2.5 cm parasagittal and before the coroner suture by themselves under instructions. Then, the ventricles were inspected with different angled optics. They then perform ETV. As a next step, a more lateral base hole was cut 4–5 cm parasagittally, and a septostomy was performed.

### Instruments

Surgical hands-on workshops require adequate equipment. The endoscope sets were provided from major companies and are comparable in size with working trocars from 4 to 6 mm diameter. The instruments were basically comparable as standard instruments like scissors, dilation forceps, grasping forceps and coagulation probes were used. However, depending on the availability of the endoscope sets variating from workshop to workshop we did not compare the differences between the different types due to the reason, that those well-established sets enable the basic steps of neuroendocopy on a similar level in our experience.

### Evaluation form

A questionnaire, as added to the supplemental section, was distributed to the participants at the end of the workshop. The questionnaire included general questions regarding age, educational level, and experience in neuroendoscopy. The model was subsequently rated concerning the handling, realism and overall learning effect. The trainees were also asked for their desired qualities of an ideal training model for neuroendoscopy. The questions were ruled out in open answers, a Likert like scale or numeric rating scale, as reported before [14].

### Statistical analysis

The questionnaires were transferred to SPSS (IBM, Armonk, USA). The results were analyzed by the chi-square test and likelihood test. A level of statistical significance was assumed at *p* ≤ 0.05. The data are presented as the mean and standard error of the mean.

## Results

Overall, 63 trainees participated in the workshop events. Twenty-six were residents, and 37 were consultants. Years of practice were categorized as follows: 1–5 years (*n* = 21); 5–10 years (20); 10–15 years (*n* = 16); and more than 15 years (*n* = 6). Twenty-eight participants had not previously undergone ETV. The remaining 35 participants had performed 1.8 ± 0.1 ETVs and were almost exclusively consultants. They frequently use neuronavigation (80%) and balloon catheters (94%). Laser techniques are only applied in 11% of cases.

A total of 97% of the participants evaluated the animal model as “very realistic” or “what is realistic” regarding surgical handling, 90% regarding anatomy, 97% regarding tissue properties and 98% in general.

The cadaver model was evaluated by 95% of the participants as “very realistic” or “what is realistic” regarding surgical handling, 100% regarding anatomy, 80% regarding tissue properties and 100% in general. These data are summarized in Table 1.

**Table 1:** Summarizes the evaluation of the realism of the murine training model and the cadaver model in terms of “handling”, “anatomy”, “tissue properties” and “overall impression”

	Murine model handling [n]	Murine model anatomic realism [n]	Murine model tissue Properties [n]	Murine model overall impression [n]	Cadaver model handling [n]	Cadever anatomic realism [n]	Cader tissue properties [n]	Cader overall impression [n]
Very realistic	35	27	36	37	24	29	13	23
Kind of realistic	24	29	24	25	14	11	19	17
More unrealistic	0	4	2	1	2	0	6	0
Unrealistic	1	1	0	0	0	0	2	0
Don’t know	4	2	1	0	0	0	0	0

The learning effect was assessed on an asset scale from low = 1 to high = 10. The animal model was evaluated with a mean score of 8.5 ± 0.2, and the cadaver model had a mean score of 9.0 ± 0.2. Residents and consultants evaluated the models nearly identically (8.4 ± 0.3 vs. 8.6 ± 0.3 and 8.9 ± 0.3 vs. 9.0 ± 0.3). There was no statistically significant difference between the groups.

The trainees were asked about their confidence in performing an ETV after performing the training model. A total of 74.0% of the participants were confident after the animal model was established, and 87.5% of the participants were confident after the cadaver model was established. After each model, the consultants felt more confident than did the residents (89% vs. 58% and 96% vs. 71%, respectively).

In addition, the trainees were asked about the requirements of an ideal training model. The participants “strongly agreed” or “agreed” in 100% with realistic simulation of anatomical structures, 98% with realistic simulation of tissue strength, 80% with realistic simulation of pulsation, 78% with realistic simulation of complications, 76% with realistic simulation of bleeding and 71% with a realistic simulation of an OR setting (Table 2).

**Table 2:** Summarizes the expected requirements for an ideal training model by the participants

	Same Instruments like in the OR [n]	Same Setting like in the OR [n]	Realistic Anatomical Structures [n]	Realistic Tissue Properties Regarding Resistance [n]	Realistic Bleeding Conditions [n]	Realistic Pulsation [n]	Simulation of Complications [n]
Strongly agree	49	17	44	41	31	23	24
Agree	14	34	19	21	17	23	19
Neither nor	0	5	0	1	5	6	6
Disagree	0	1	0	0	4	5	6

## Discussion

This study showed that a murine model can achieve training effects comparable to those of cadaveric models for neuroendoscopic procedures. More experienced surgeons feel comparable confidence in performing an ETV after being trained in such a model in comparison to a cadaver hands-on session. Young colleagues also rated the model as an adequate learning method. Nonetheless, the study is limited by the subjective questions in the questionnaire. There remains always the risk for positive bias. Objective assessments of the task and learning effects can overcome this problem in future studies. The strength of the murine models can be seen in its simulation of a live surgery with the immediate feedback of actions and failures. It puts the trainee in a stressful situation and emphasizes the consequences of his or her actions. The surgical task can be performed in under 30 min, which enables a sufficient time plan during a two-day workshop, for example. A limitation of the murine model is surely that it cannot help to teach the intra- and paraventricular anatomy of the human brain. Another limitation might be the indispensable infrastructure, that is necessary to obtain the justified standards to perform animal models. Nonetheless, sacrificing animals for educational reasons has to be discussed. Individual trainees might be uncomfortable with this task for ethical reasons and this decision has to be respected. Ongoing developments of alternative training models might overcome the need of animal models, overall.

The overall cost of a single rat is cheaper compared to a human cadaver model. A facility for animal experiments is mandatory of course. Fixed or fresh frozen cadavers are also not overall available in many countries. Those cadavers are also single use, as the introduction of the endoscope and ETV can be performed only once. Especially fresh frozen cadaver suffer from an insufficient consistency of the brain tissue and adequate puncture of the ventricle and maneuvering is common. Without neuronavigation the trainees have often frustrating attempts to enter the ventricle and may destroy parts of the anatomy.

Simulating the anatomy was one of the most important requirements for an ideal model in our poll. Other training models may overcome this problem, as cadaver models are not ubiquitously available all over the world or are restricted by shortages or low prices. 3D-printed models are helpful for teaching anatomy and might help to encourage medical students to choose neurosurgery at all; however, training manual skills effectively is doubtable [14, 15].

Various computer simulations have been developed and have shown fair results concerning anatomical learning effects. Nonetheless, as this technology was promoted more than 15 years ago, it is still not routinely implemented [16]. A major drawback of virtual reality is the inadequate simulation of force sensing and tissue properties. The importance of this simulation aspect has also been shown in our evaluation. Emerging technologies might overcome this problem in the near future toward establishing low-cost, high-performance MIS force sensors, such as promising piezoelectric sensors. Recent rapid advances in computer vision and machine learning have drawn increasing attention to imaging-based tactile sensing, also known as vision-based sensing [17]. Augmented or virtual reality will continue to result in rapid advancements in operative planning, intraoperative navigation, and neurosurgical training [18].

Interestingly, complication simulation was only important for 76% of all trainees. In our opinion, this aspect is common in neuroendoscopic intraventricular procedures, as complications are major risks for patients [8, 10]. In particular, hemorrhages might be challenging to handle and need immediate proper measures [19].

Models could be improved by testing the effects on the manual skills and knowledge of the participants. However, standardized evaluation methods are lacking and should be developed by surgical societies regardless of the proper model type [20].

## Data Availability

No datasets were generated or analysed during the current study.
